# Case series of diagnosis and surgery challenges in parathyroid carcinoma

**DOI:** 10.1016/j.ijscr.2022.107390

**Published:** 2022-07-09

**Authors:** Diani Kartini, Ahmad Kurnia, Erwin Danil Yulian, Sonar Soni Panigoro, I. Gusti Ngurah Gunawan Wibisana, Jessica Wardana

**Affiliations:** Division of Oncology Surgery, Department of Surgery, Faculty of Medicine Universitas Indonesia, Dr. Cipto Mangunkusumo General Hospital, Jakarta, Indonesia

**Keywords:** 4D-CT, 4-dimensional computed tomography, MRI, magnetic resonance imaging, PA, parathyroid adenoma, PC, parathyroid carcinoma, PTH, parathyroid hormone, US, ultrasound, Case series, En bloc resection, Ipsilateral parathyroidectomy, Hyperparathyroidism, Parathyroid carcinoma, Parathyroidectomy

## Abstract

**Introduction:**

Parathyroid carcinoma (PC) is a rare malignancy that accounts for 1 % of cases of hyperparathyroidism. Data regarding PC in Indonesia are scarce, which poses challenges to diagnosis and treatment. This study aims to describe a series of PC cases from a tertiary health care center over 12 years.

**Presentation of cases:**

Retrospective data of six patients with hyperparathyroidism diagnosed with PC between 2008 and 2020 were reviewed. Clinical presentation, diagnosis, management, and short-term outcomes of PC were analyzed. All six PC patients were diagnosed postoperatively. Four of the patients presented with symptomatic hypercalcemia, and two presented with neck swelling. Elevated serum parathyroid hormone was observed in five patients. Only two patients had imaging results corresponding to PC characteristics. Ipsilateral parathyroidectomies were performed on 5 patients where invasion and metastasis are not evident. Four frozen section samples suggested PC, and two suggested parathyroid adenoma. Further histopathologic examination confirmed a diagnosis of PC in all patients. No metastasis to the adjacent lymph nodes or distant target organs was found during surgery.

**Discussion:**

Preoperative diagnosis of PC remains challenging. Suspicion of PC is appropriate in the presence of severe hypercalcemia, elevated parathyroid hormone level, and a mass observed either during imaging or intraoperatively.

**Conclusion:**

Ipsilateral parathyroidectomy seems to be feasible compared to total resection in order to preserve function and structure. Incomplete excision may lead to an increased risk of recurrence, emphasizing the importance of routinely following up on PC cases.

## Highlights

• Parathyroid carcinoma is a very rare endocrine neoplasm.
• Severe hypercalcemia (>14 mg/dL), high PTH (3 to 10 times the upper limit of normal), and a neck mass raise suspicion for PC
• No specific guideline or consensus existed
• Very similar presentation with parathyroid adenoma
• Ipsilateral parathyroidectomy deemed feasible for parathyroid carcinoma without signs of malignancy and invasions

## 1 Introduction

Parathyroid carcinoma (PC) is one of the rarest endocrine malignancy [1]. PC is clinically difficult to distinguish from benign causes of primary hyperparathyroidism (PHPT). Reports have recognized PC cases in multiple endocrine neoplasia 1 (MEN1), an autosomal dominant syndrome affecting endocrine tumors. 90 % of MEN1 patients found presented with hyperparathyroidism [2]. Primary hyperparathyroidism is usually characterized by hypercalcemia, which occurs due to excessive autonomous secretion of parathyroid hormone (PTH) and is three to four times more likely to be found in women aged 50 to 65 years [3], [4].

PC is indicated by a mass with signs of malignancy observed by imaging or a tender mass with uneven margins found intraoperatively. Findings of serum calcium levels higher than 14 mg/dL and PTH levels 10 times or more than the upper limit of normal indicate malignancy [5]. The absolute prerequisite for PC consists of lymph node metastasis, local invasion, and distant metastases [6], [7]. Histopathologic examination remains the gold standard and the only method to establish the diagnosis of PC [8].

Complete surgical resection of thyroid and parathyroid glands (en bloc resection) with microscopically negative margins is the gold standard for PC treatment [1]. Less radical resection may lead to a higher risk of recurrence [9]. Radiation therapy and chemotherapy have not been proven beneficial in PC management [1], [10], [11]. Data regarding PC profiles in Indonesia are still limited. This case series aims to describe the clinical manifestations, diagnosis, management, and outcome of PC over 12 years from a single institution.

## 2 Presentation of cases

This was a single retrospective study and case series of all patients with hyperparathyroidism treated in the Surgical Oncology Clinic of Cipto Mangunkusumo General Hospital, Jakarta, between January 1, 2008, and December 31, 2020. Medical records of patients diagnosed with PC postoperatively by histopathologic examination were reviewed. We obtained written informed consent from all patients. This case series has been reported in line with the PROCESS Guideline [12].

Six patients were identified, with a median age of 29.5 years (28–52 years), four were female and two were male. Skeletal involvements were found in 50 % of cases, while renal and gastrointestinal involvements were each present in 16.7 % of cases. Neck swelling was observed in two patients, and four patients had hypercalcemic symptoms, consisting of gastrointestinal complaints, bone pain, and reduced urine volume. Hypercalcemia was biochemically evident in three patients, although all patients had symptoms of hypercalcemia: bone fracture, joint stiffness, and painless lumps. One patient with a history of routine hemodialysis due to chronic renal failure had a brown tumor in his mandible and nephrolithiasis. The median serum calcium level at presentation was 13.8 mg/dL (8.5–16.7 mg/dL). Elevated serum PTH was noted in five patients preoperatively, with a median of 1593 pg/mL (82.5–2898 pg/dL). All patients in our case series underwent neck US examination. Characteristics of PC were revealed in two patients. Sestamibi scan was performed in two patients, both yielded findings suggestive of PA.

Surgery was performed by our attending surgical oncologists. One patient had en bloc resection and five had ipsilateral parathyroidectomy. None of the patients received pre-intervention treatment as there were no clinical indications. Intraoperative findings were consistent with PC characteristics, specifically, a firm, gray-colored mass with gross adherence to adjacent tissues. Frozen section samples were taken from all patients during surgery, and four of six samples exhibited PC characteristics. Histologically, all six patients showed characteristics corresponding to PC. Only one patient was observed with perineural and lymphovascular invasion. Neither metastases nor surgical complications were reported. No patients received adjuvant radiotherapy. At present, five patients are known to be alive and one patient is deceased (Table 1).

**Table 1:** Clinicopathologic, laboratory and imaging features of six PC patients.

Sex	Age	Clinical symptoms	Calcium level (mg/dL) [N: 8.4–10.2]		PTH level (pg/mL) [N: 15–65]			Preoperative imaging	Frozen section	Treatment
			Pre	Post	Pre	Intra	Post			
Male	46	Painless lump on upper mouth palate, history of routine hemodialysis due to chronic renal failure	8.5	N/A	2898	2607	–	US: Hypoechoic lesion at posterior left thyroid, possible parathyroid origin. dd/PA 4D-CT: Oval-shaped hypodense lesion at bilateral posteroinferior thyroid lobes. The right lobe was not well circumscribed, the left lobes were well circumscribed. dd/PA. Multiple lytic lesions and thinning of the cortex were observed in the craniofacial bones, mainly the maxilla and mandibula. Signs of osteopenia were also found	Parathyroid mass was histologically appropriate for parathyroid hyperplasia (left inferior lobe) and carcinoma (right lobe)	Ipsilateral parathyroidectomy
Male	28	Lump on the left thigh for 4 months	16.6	6.8	1872	–	8.51	US: Solid tumor likely to be malignant, possibly originating from the right parathyroid lobe. Lymphadenopathy was observed at the submandibular and superior right neck MRI: No focal lesion or enhancement observed in the right thyroid bed. Bilateral neck lymphadenopathy was not found Sestamibi: Mass on left parathyroid lobe suggestive of adenoma; no remaining mass on right thyroid and parathyroid lobe was observed	Malignant tumor supporting PC diagnosis	Ipsilateral parathyroidectomy
Female	26	Neck lump, complaints of bone fracture	13.4	8.9	–	–	202.7	US: Morphology of bilateral thyroid lobes was within normal limits; no mass was found in both parathyroid lobes. No lymphadenopathy was observed MRI: Left neck mass suggestive of a parathyroid tumor with intrathoracic extension. No tumor infiltration into the thyroid gland, trachea, or vascular structures of the neck was observed. No lymphadenopathy of the neck was observed	Benign parathyroid lesion, suggestive of hyperplasia	Ipsilateral parathyroidectomy
Female	31	Coccygeal stiffness for 7 months	12.9	9.3	1327	–	34.91	US: Left supraclavicular lesion was found, suggestive of lymphadenopathy. Bilateral thyroid lobes were within normal limits MRI: No lesion or lymph node enlargement was observed at the neck. Thyroid and parathyroid glands were within normal limits, and no enlargement of the parathyroid gland was observed Sestamibi: Imaging suggestive of PA at the inferior lobe was observed	Frozen section indicative of PA; lymph node metastasis was not found in preparation	Ipsilateral parathyroidectomy
Female	24	Recurring nausea and vomiting in the last 1 year	14.2	7.8	82.51	–	–	US: Left thyroid struma was observed, and a hypoechoic mass of the posterior thyroid lobe suggestive of PA was also observed MRI: Left parathyroid mass with a measurement of ±3.05 × 2.61 × 2.47 cm	Histologically appropriate for PC	Ipsilateral parathyroidectomy
Female	52	Lump on left side of the neck for 10 months	16.7	13.8	1593	–	124	–	Histologically appropriate for PC	En bloc resection

Abbreviations: 4D-CT, 4-dimensional computed tomography; dd, differential diagnosis; MRI, magnetic resonance imaging; PA, parathyroid adenoma; PC, parathyroid carcinoma; PTH, parathyroid hormone; US, ultrasound.

## 3 Discussion

Aside from the female predominance, our patients had a younger demographic compared with most studies. A study of 330 PC cases showed a mean and median age of 49 years, whereas the median age of our patients was 29.5 years [3]. Neck mass often indicates malignancy, as it is rarely seen in benign cases. Only 10 % of PCs are nonfunctioning, with patients complaining solely of neck mass and no other symptoms of organ involvement [3].

Suspicion of PC should arise in patients with serum calcium levels higher than 14 mg/dL and PTH levels 3 to 15 times the upper limit of normal. Two patients were indicative of PC because of their elevated serum calcium and PTH levels. Four patients were not biochemically suggestive of malignancy, because their serum calcium levels is lower than 14 mg/dL, even though their PTH levels were extremely high. This finding highlights the existence of a wide overlap between benign and malignant tumors when viewed from a biochemical perspective.

Imaging studies were fundamental before surgery. Our study utilized neck US as part of the routine examination. Two of six PC cases were identified by neck US, while two patients who underwent MIBI scan yielded findings suggestive of PA. Localization may be aided by MIBI scan, but no sole imaging modality has been fully validated for the detection of PC [4]. Imaging must be performed in conjunction with biochemical profiles and histopathologic findings.

Frozen section was our routine procedure for histopathologic sampling. Two of the six cases were determined to have malignant characteristics according to frozen section samples, although pathologic examination revealed all six to be PC. Fine needle aspiration biopsy (FNAB) was not considered, as the risk of tumor rupture and seeding may increase the likelihood of recurrence [13].

In this study, only one patient underwent en bloc resection because we found macroscopic adherence of the tumor to adjacent tissues and signs of malignancy in its immediate-read frozen section. In most of our cases, we performed ipsilateral parathyroidectomy because there were no signs of adhesions or invasion of surrounding tissue, including thyroid tissue, was seen during surgery. The histopathologic results of all patients corresponded with criteria for PC although only one sample showed vascular and capsular invasion.

Recurrences usually occur within 2 to 3 years after the initial surgery, mostly within the regional operative field, and are reflected by marked hypercalcemia and hyperparathyroidism identified at follow-up [4]. Surgery is rarely curative in cases with distant metastases, although palliative debulking of the tumor mass may aid in controlling hypercalcemia [11]. In the largest cohort evaluating the overall survival of PC patients, no significant difference was found between en bloc resection and local resection [14]. Radical en bloc resection is controversial, because it leads to significant morbidities, such as muscular dysfunction and laryngeal nerve palsies [14], [15]. Locoregional surgeries, including ipsilateral parathyroidectomy, were found useful in certain situations, such as local disease control, when a less aggressive approach was opted for. However, more studies reported that patients with ipsilateral parathyroidectomy were found to undergo en bloc resection to achieve disease control and eliminate residual disease [15], [16], [17], [18]. Therefore, en bloc resection was still deemed preferable to avoid reoperations and achieve remission.

Neither radiotherapy nor chemotherapy was done in this study because PC is considered radioresistant. Although a few small retrospective studies have found a lower recurrence rate with adjuvant radiotherapy [7], [9]. Successful chemotherapy has been reported, but data on the efficacy of adjuvant chemotherapy are still lacking [5]. Long-term outpatient management is commonly required in which patients are treated with calcimimetic therapy with or without bisphosphonate therapy to aid in decreasing calcium levels and associated symptoms [9]. Morbidity and mortality of PC patients are largely due to sequelae of hypercalcemia and end-organ damage [5].

Our study strengths are the inclusivity of all PC patients over years and comparison of clinical evaluations made to decide the method of surgery opted. We also showed that ipsilateral parathyroidectomy could be considered in some cases compared to en bloc resection. The study limitations include the absence of follow-up due to nonattendance during COVID-19 pandemic, which prevents observation of recurrence of the disease and performance of re-exploration for those receiving only ipsilateral parathyroidectomy. Follow-up should be lifelong, with measurement of calcium and PTH levels every 6 months [10].

The prognosis of PC is dependent on complete resection at initial surgery, although a prognostic classification system developed by Talat and Schulte may be used to determine patient survival [19]. To date, there is no consensus or guidelines for PC due to the rarity of the disease. Studies usually consisting of case reports and case series have too few cases to represent the general population. Further studies involving a large number of patients are warranted to establish a general guideline for PC.

## 4 Conclusion

Parathyroid carcinoma is a rare disease which often mimics its benign counterparts posing a challenge for diagnosing PC preoperatively. Suspicion of PC is appropriate in the presence of severe hypercalcemia (>14 mg/dL), high level of PTH (3 to 10 times the upper limit of normal), and a large mass, observed either during imaging or intraoperatively. The best chance for a cure is complete surgical resection, which can be achieved by en bloc resection. However, whether to perform this extensive surgical procedure or local resection by ipsilateral parathyroidectomy is a difficult decision determined by patients' condition and tumor adherence. Incomplete excision may lead to an increased risk of recurrence, thus emphasizing the importance of close follow-up of PC cases that could last for a lifetime.

## Provenance and peer review

Not commissioned, externally peer-reviewed.

## Ethical approval

The study was exempted from ethical approval in our institution, Faculty of Medicine Universitas Indonesia because it is a retrospective study with data taken solely from medical records.

## Funding

None.

## Guarantor

Diani Kartini, as the guarantor, hold full responsibility for the work and conduct to the study and hold the decision to publish.

## Research registration number

-

## CRediT authorship contribution statement

**Diani Kartini:** Conceptualization, Methodology, Validation, Formal analysis, Investigation, Resources, Writing – Review and editing, Supervision.
**Ahmad Kurnia:** Conceptualization, Methodology, Validation, Formal analysis, Investigation, Resources, Writing – Review and editing,
**Erwin Danil Yulian:** Conceptualization, Methodology, Validation, Formal analysis, Investigation, Resources, Writing – Review and editing,
**Sonar Soni Panigoro:** Conceptualization, Methodology, Validation, Formal analysis, Investigation, Resources, Writing – Review and editing,
**I Gusti Ngurah Gunawan Wibisana:** Conceptualization, Methodology, Validation, Formal analysis, Investigation, Resources, Writing – Review and editing,
**Jessica Dewati Wardana:** Resources, Writing – Original draft, Writing – Review & editing.

## Declaration of competing interest

The authors declare they have no conflicts or financial ties to disclose.
